# Passive Ankle Dorsiflexion and Single-Leg Balance Are Independently Associated with Locomotive Syndrome Severity in Community-Dwelling Older Adults: A Cross-Sectional Study

**DOI:** 10.3390/healthcare14060742

**Published:** 2026-03-14

**Authors:** Satoshi Hakukawa, Junpei Matsumoto, Yusuke Kawamura

**Affiliations:** 1 Department of Rehabilitation, Reiwa Rehabilitation Hospital, Chibaminato 4-4, Chuo Ward, Chiba 260-0026, Chiba, Japan; 2 Department of Rehabilitation, Kiminomori Orthopaedics Clinic, Kiminomoriminami 1-30-5, Oamishirasato 299-3241, Chiba, Japan; 3 Department of Home Care Support, Kiminomori Rehabilitation Hospital, Kiminomoriminami 1-30-1, Oamishirasato 299-3241, Chiba, Japan

**Keywords:** locomotive syndrome, ankle dorsiflexion, single-leg stance, hallux valgus, navicular height, community-dwelling older adults

## Abstract

**Highlights:**

**What are the main findings?**

- Reduced passive ankle dorsiflexion and poorer single-leg balance were independently associated with greater locomotive syndrome severity in community-dwelling older adults.
- Static foot morphology measures (hallux valgus angle and navicular height) were not independently related to locomotive syndrome severity.

**What are the implications of the main findings?**

- Simple assessments of ankle dorsiflexion and single-leg balance may aid screening and early risk stratification for locomotive syndrome in community settings.
- Interventions targeting ankle dorsiflexion and balance warrant prospective evaluation to prevent progression of mobility decline.

**Abstract:**

**Background/Objectives**: Foot impairments are common in older adults, but the independent associations of specific foot indices with locomotive syndrome (LS) severity remain unclear. We examined hallux valgus angle (HV), navicular height (NH), and passive ankle dorsiflexion (ADF). **Methods**: This cross-sectional study included 119 community-dwelling older adults classified into LS stages 0–3. Bilateral measures were summarized as maximum HV and minimum NH/ADF, reflecting the worst-affected side. Proportional-odds ordinal logistic regression modeled LS stage (0–3) with foot indices and covariates (age, sex, body mass index [BMI]). Extended models additionally adjusted for Timed Up and Go (TUG), gait speed, or single-leg stance (SLS). Sensitivity analysis used binary logistic regression (LS ≥ 2 vs. <2). **Results**: Greater ADF was independently associated with lower LS severity (OR per 1°, 0.91; 95% CI, 0.85–0.98; *p* < 0.01), whereas higher BMI was associated with greater LS severity (OR per 1 kg/m^2^, 1.15; 95% CI, 1.01–1.30; *p* < 0.05). HV and NH were not significant. After adjustment for TUG, gait speed, or SLS, ADF remained inversely associated with LS severity (ORs, 0.92–0.93; *p* < 0.05), while the BMI association was attenuated. In binary logistic regression, greater ADF was associated with lower odds of LS ≥ 2 (OR per 1°, 0.85; 95% CI, 0.76–0.94; *p* < 0.005). **Conclusions**: Reduced passive ankle dorsiflexion is independently associated with greater LS severity, robust after accounting for key mobility and balance measures. Interventions targeting ankle mobility may represent a potentially modifiable factor and warrants confirmation in longitudinal and interventional studies.

## 1. Introduction

In older adulthood, age-related declines in muscle mass and strength, alterations in neuromuscular control, and the emergence of joint range-of-motion limitations and pain can destabilize fundamental activities of daily living such as walking and rising from a chair, thereby increasing the likelihood of reduced mobility. Such mobility decline may lead to less frequent outings and lower physical activity, which in turn can accelerate physical deconditioning and restrict social participation, ultimately increasing the risk of requiring long-term care—an important clinical and public health concern [1]. In Japan, the concept of locomotive syndrome (LS) was proposed to describe a condition in which impairments of the musculoskeletal system cause difficulties in walking and daily activities and are associated with a high risk of needing nursing care [2]. LS has since been positioned as a framework that links musculoskeletal disorders to functional decline and promotes early detection and timely intervention [3]. To assess LS, the Locomotive Syndrome Risk Test—which combines functional tests (the stand-up test and the two-step test) with a questionnaire, the 25-item Geriatric Locomotive Function Scale (GLFS-25)—has become widely used in both clinical and community settings as a quantitative approach to evaluating mobility decline. Moreover, following a recent revision of the diagnostic criteria, LS stage 3 was introduced to distinguish individuals with more severe restrictions in social participation according to disease severity [4]. Importantly, the GLFS-25 has been translated across multiple languages and cultural contexts, supporting its international applicability and comparability beyond Japan. For example, validated versions have been reported in Turkish and Brazilian Portuguese populations [5,6].

LS is important not merely as an indicator of “physical deconditioning” but because it can contribute to progressive mobility decline through bidirectional interactions with musculoskeletal disorders such as osteoarthritis, spinal disorders, osteoporosis, and sarcopenia. The pain, malalignment, and impaired joint function associated with these conditions may alter gait patterns and compromise balance, thereby increasing the risk of falls and fractures and ultimately leading to declines in activities of daily living (ADL) and quality of life (QOL). Indeed, LS has been reported to be associated with fall and fracture risk [7,8], suggesting that preventing progression to more severe LS may be meaningful from the perspectives of fall prevention and long-term care prevention. LS has also been linked to poorer health-related quality of life (HRQOL), and accumulating evidence has examined associations between LS and pre-frailty, as well as relationships with QOL measures such as the EuroQol 5-Dimension (EQ-5D) [9,10]. Therefore, LS may serve as a clinically relevant outcome that captures the pathway from “musculoskeletal functional impairment” to “decline in daily functioning” and represents an implementable target for early interventions aimed at maintaining functional independence.

However, mobility is not determined solely by proximal factors such as muscle strength and joint function of the knee, hip, or spine; it is also strongly influenced by the foot, which directly contacts the ground during gait and plays a central role in load-bearing and propulsion. With aging, the prevalence of reduced foot arch height, toe deformities, skin problems (e.g., calluses), and foot pain is known to increase [11,12]. These foot-related problems may affect weight-bearing capacity, load distribution, propulsion generation, and sensory input, thereby impairing gait and balance [13,14]. In the Framingham Foot Study, foot pain was independently associated with mobility limitation, suggesting that foot symptoms themselves may be an important determinant of reduced activity in older adults [15]. In the same cohort, in addition to foot pain, foot alignment and function—such as a planus foot posture and pronated foot function—were reported to be associated with self-reported mobility limitation, supporting the possibility that foot-related indices and function contribute to mobility decline [16].

Moreover, studies in community-dwelling older adults have shown that measures such as foot/ankle mobility and foot deformities are associated with balance and functional test performance [17]. In particular, older adults with toe deformities, including hallux valgus, have been reported to exhibit different gait and balance characteristics, and foot arch morphology (e.g., arch height and arch stiffness) may also be related to postural control [18]. Ankle dorsiflexion range of motion (ROM) has likewise been linked to balance ability [19]; when dorsiflexion is limited, control of the center of mass in standing and forward progression of the tibia during the stance phase may be constrained, potentially disadvantaging performance in mobility-related tasks.

However, the accumulation of evidence regarding how these foot-related indices are associated with LS remains insufficient. Although one cross-sectional study has reported an association between foot arch morphology and locomotive function-related indicators [20], few studies in community-dwelling older adults have comprehensively examined the relationship between LS and multiple, relatively feasible foot measures—such as hallux valgus, arch height, and ankle dorsiflexion—while simultaneously evaluating physical function indicators including gait and balance. Linking the LS assessment framework with foot-related indices and functional measures may help clarify modifiable factors that contribute to mobility decline.

Therefore, this study aimed to investigate the associations between LS and readily measurable foot-related indices—hallux valgus (HV), medial longitudinal arch height (AH), and ankle dorsiflexion angle (ADF)—in community-dwelling older adults, while also assessing physical function (e.g., gait and balance). We hypothesized that individuals with more severe HV, lower AH (i.e., morphologically disadvantageous arch structure), and smaller ADF would demonstrate poorer physical function, which in turn would be associated with a higher risk or greater severity of LS. Given prior evidence linking ankle dorsiflexion with postural control and gait stability, we further expected that limited ADF would show a relatively clear association with impaired physical function and, consequently, with LS.

## 2. Materials and Methods

### 2.1. Participants

This cross-sectional study included community-dwelling older adults aged ≥60 years who were enrolled in 2025. Participants were recruited as community volunteers through community-based health events (local health checkups and fitness assessment sessions) and posted announcements, using convenience sampling. The inclusion criterion was the ability to ambulate independently without assistance. Exclusion criteria were suspected cognitive impairment that could interfere with assessment, a history within the previous 6 months of fractures or neurological disorders that could affect gait, and missing data. The study protocol was approved in advance by the institutional ethics committee. Written informed consent was obtained from all participants prior to enrollment, including consent for publication of the study results.

### 2.2. Locomotive Syndrome Assessment

To classify LS stage, the Locomotive Syndrome Risk Test was administered, comprising the stand-up test, the two-step test, and the 25-item Geriatric Locomotive Function Scale (GLFS-25). For the stand-up test, all participants were first confirmed to be able to stand up from a 40 cm seat height using both legs; they were then assessed for the ability to stand up from 40 cm using one leg. If a one-leg stand-up from 40 cm was not possible, participants attempted a two-leg stand-up from 30 cm and subsequently from 20 cm. Based on stand-up performance, participants were categorized as non-LS (one-leg stand-up from 40 cm), LS stage 1 (LS1) (unable to perform a one-leg stand-up from 40 cm but able to stand up from 20 cm using both legs), LS stage 2 (LS2) (unable to stand up from 20 cm but able to stand up from 30 cm using both legs), or LS stage 3 (LS3) (unable to stand up from 30 cm using both legs). For the two-step test, participants took two maximal strides forward from a starting line, and the total distance from the starting line to the tip of the toe at landing was measured (cm). The two-step score was calculated as the total distance covered in two steps (cm) divided by the participant’s height (cm), and participants were categorized as non-LS (score ≥ 1.3), LS1 (1.1–<1.3), LS2 (0.9–<1.1), or LS3 (<0.9). The GLFS-25 is a self-administered questionnaire consisting of 25 items (4 pain-related items, 16 items on activities of daily living, 3 items on social function, and 2 items on mental health), each rated on a 5-point Likert scale from 0 (no impairment) to 4 (severe impairment). The total score (range, 0–100) was calculated as the sum of all items, with higher scores indicating greater locomotive dysfunction; participants were categorized as non-LS (<7), LS1 (7–<16), LS2 (16–<24), or LS3 (≥24). The final LS stage was determined as the most severe classification (i.e., the highest stage) obtained across the three assessments.

### 2.3. Foot-Related Measurements

Foot-related indices included the hallux valgus angle (HV), navicular height (NH), and passive ankle dorsiflexion angle (ADF). HV and NH were measured from photographs taken in a standardized standing position. Although weight-bearing radiography is considered the reference standard for evaluating hallux valgus deformity, radiographic assessment was not feasible in our community-based field setting due to radiation exposure, cost, and equipment constraints. Therefore, we used standardized digital photographs; photographic measurements of hallux valgus angle have shown high reliability and acceptable concurrent validity compared with weight-bearing radiographs [21]. NH is a non-invasive clinical indicator of medial longitudinal arch structure, and standardized external measurements have demonstrated acceptable reliability in clinical settings [22]. In addition, digital photographic approaches have been reported to provide valid and reliable quantification of arch- and navicular-related structural parameters under standardized procedures [23], supporting their use in field-based assessments. ADF was measured from photographs obtained in the supine position while the examiner passively moved the ankle to maximal dorsiflexion. During passive ADF measurement, the knee was maintained in full extension to standardize gastrocnemius tension. A weight-bearing lunge test may better reflect functional dorsiflexion, but it can be substantially affected by pain, balance, lower-limb strength, and movement strategy, which may vary across individuals and confound comparisons in a field-based assessment [24]. Therefore, we selected a standardized passive protocol as a feasible and safer approach for community measurements, while acknowledging that passive ADF may not fully represent dynamic weight-bearing dorsiflexion. All measurements were performed using ImageJ (version 1.54h; National Institutes of Health, Bethesda, MD, USA) by two physical therapists with >10 years of clinical experience. Measurements were obtained bilaterally. Because bilateral foot impairments are common and may be asymmetric, we prespecified a “worst-side” approach to derive representative values: the maximum HV and the minimum NH and ADF, reflecting the side with the greatest deformity or restriction that is most likely to limit weight-bearing function. As a sensitivity analysis, we repeated the primary models using averaged bilateral values to assess robustness.

### 2.4. Physical Function Assessment

Physical function was assessed using gait speed, the 3 m Timed Up and Go test (TUG), and single-leg stance time (SLS). Gait speed was calculated during a 7 m walk at a comfortable pace using the central 5 m, excluding the first and last 1 m for acceleration and deceleration. The TUG measured the time required to complete a sequence of standing up, forward walking, turning, return walking, and sitting down; participants performed one trial each with right and left turns at maximal speed [25]. For SLS, participants stood on one leg with their hands on their hips, ensuring that the legs did not touch, for up to a maximum of 60 s. Each test was performed twice, and the better performance was used for analysis.

### 2.5. Statistical Analysis

All statistical analyses were conducted in R (version 4.5.2; R Foundation for Statistical Computing, Vienna, Austria) using the R Commander graphical user interface. Prior to inferential testing, distributions of continuous variables were inspected for normality, skewness, and outliers using the Shapiro–Wilk test and Q-Q plot. Continuous variables are summarized as mean ± standard deviation when approximately normally distributed and as median [interquartile range] otherwise, and categorical variables are summarized as numbers (%). To describe differences across the four LS stages (0–3), we used one-way analysis of variance (ANOVA) or the Kruskal–Wallis test for continuous variables and the chi-square test for categorical variables (and Fisher’s exact test when expected cell counts were small). When an overall group difference was detected, post hoc comparisons were performed using Tukey’s test following ANOVA or Dunn’s test following the Kruskal–Wallis test, with multiplicity controlled using the Holm method.

For the primary analysis, because LS stage (0–3) is an ordinal outcome, we employed multivariable ordinal logistic regression under a proportional-odds framework to examine whether foot-related indices were independently associated with LS severity. The foot-related indices were hallux valgus angle (HV), navicular height (NH), and passive ankle dorsiflexion (ADF), each measured bilaterally. As prespecified, the maximum HV and the minimum NH and ADF were used as representative values, reflecting the potential clinical relevance of the more deformed or restricted side. Age, sex (coded as 0 = female and 1 = male), and body mass index (BMI) were included as covariates. Results are reported as odds ratios (ORs) with 95% confidence intervals (CIs), where ORs represent the odds of being classified into a higher (more severe) LS stage per 1-unit increase in each predictor.

To support the use of the proportional-odds model, we evaluated the proportional-odds assumption by comparing the ordinal model with a multinomial logistic regression model using a likelihood-ratio test. In addition, to explore whether physical function could account for the association between foot-related indices and LS severity, we fitted extended ordinal logistic regression models by adding physical function measures to the primary model one at a time: TUG, gait speed, and SLS. For these extended models, changes in effect estimates for the foot-related indices and model fit were examined using likelihood-ratio tests for nested models and Akaike’s information criterion (AIC).

Sensitivity analyses were conducted to assess the robustness of the primary findings. First, we fitted a binary logistic regression model using dichotomized LS (LS ≥ 2 vs. <2). Multicollinearity among predictors was assessed using the variance inflation factor (VIF). Second, as exploratory analyses, we examined associations between foot-related indices and individual components of the LS classification (two-step test, stand-up test, and the 25-question Geriatric Locomotive Function Scale [GLFS-25]) using Spearman’s rank correlation coefficients and partial Spearman correlations adjusted for age, sex, and BMI. These analyses were considered exploratory and are presented in the Supplementary Materials. All tests were two-sided, with *p* < 0.05 indicating statistical significance. Because this study was exploratory and field-based, we did not perform an a priori sample size calculation. Therefore, the study may be underpowered to detect small effects and to support analyses stratified by LS stage, particularly when some outcome categories are sparsely populated. We provide a post hoc sensitivity analysis using G*Power version 3.1.9.6 (Heinrich-Heine-Universität Düsseldorf, Düsseldorf, Germany) based on the available sample size (n = 119) to aid interpretation.

## 3. Results

### 3.1. Participant Characteristics by LS Stage

A total of 119 participants were included in the analyses: non-LS (LS0), n = 24; LS1, n = 73; LS2, n = 13; and LS3, n = 9. In the sensitivity analysis using G*Power based on the available sample size (n = 119), assuming six predictors, a two-sided alpha of 0.05, and 80% power, the minimum detectable effect size was Cohen’s f^2^ = 0.121 (corresponding to a minimum R^2^ = 0.108). Age did not differ significantly in groups with more severe LS (*p* = 0.051). There were no significant between-group differences in sex, BMI, HV, or NH (*p* = 0.937, 0.159, 0.484, and 0.614, respectively). In contrast, ADF was smaller in groups with more severe LS, showing a significant between-group difference (*p* = 0.001). Comfortable gait speed and maximal speed TUG time were poorer (slower gait speed and longer TUG time) with increasing LS severity, with significant between-group differences for both (*p* = 0.001 for each). SLS time also differed significantly across groups (*p* = 0.001) (Table 1).

### 3.2. Associations Between Foot-Related Indices and LS Stage (Ordinal Logistic Regression)

We performed proportional-odds ordinal logistic regression with LS stage (0–3) as the dependent variable, including foot-related indices (HV, NH, and ADF) and covariates (age, sex coded as 0 = female and 1 = male, and BMI). The primary model showed a significantly better fit than the intercept-only model (LR χ^2^(6) = 20.53, *p* = 0.002). In the multivariable model, higher BMI was independently associated with greater LS severity (OR per 1 kg/m^2^, 1.15; 95% CI, 1.01–1.30; *p* = 0.038), whereas greater ADF was independently associated with lower LS severity (OR per 1°, 0.91; 95% CI, 0.85–0.98; *p* = 0.008). HV and NH were not significantly associated with LS stage (both *p* > 0.27). Sex and age did not reach statistical significance (sex: *p* = 0.092; age: *p* = 0.082). Model fit indices were residual deviance = 186.72 and AIC = 204.72 (Table 2). The proportional-odds assumption was not rejected (likelihood-ratio test versus multinomial model, *p* = 0.675), supporting the use of the proportional-odds model.

In an extended model additionally adjusting for TUG, model fit further improved compared with the intercept-only model (LR χ^2^(7) = 34.97, *p* < 0.001; AIC = 192.27). Longer TUG time was strongly associated with greater LS severity (OR per 1 s, 2.34; 95% CI, 1.48–3.70; *p* < 0.001). Importantly, ADF remained inversely associated with LS severity after adjusting for TUG (OR per 1°, 0.92; 95% CI, 0.86–0.99; *p* = 0.015), whereas BMI was no longer significant (*p* = 0.748). Similar patterns were observed in extended models adjusting for comfortable gait speed and single-leg stance time (Supplementary Table S1).

### 3.3. Sensitivity Analysis (Binary Logistic Regression)

We performed multivariable binary logistic regression comparing LS ≥ 2 versus <2 (n = 119), including HV, NH, ADF, BMI, sex, and age. The full model fit significantly better than the intercept-only model (likelihood-ratio χ^2^(6) = 24.87, *p* < 0.001). Greater passive ADF was independently associated with lower odds of LS ≥ 2 (OR per 1°, 0.85; 95% CI, 0.76–0.94; *p* = 0.002), whereas HV and NH were not significant (*p* = 0.854 and *p* = 0.461, respectively). Multicollinearity was not a concern in the primary model (VIF; range: 1.065 to 1.431). BMI and sex showed non-significant trends (*p* = 0.076 and *p* = 0.090) (Supplementary Table S2). Exploratory partial Spearman correlations between foot-related indices and LS component tests are provided in Supplementary Table S3.

## 4. Discussion

This study examined associations between foot-related indices (HV, NH, and ADF) and LS in community-dwelling older adults. In the four-group comparison by LS stage, participants with more severe LS exhibited smaller ADF, with significant between-group differences. Moreover, in multivariable analyses adjusted for age, sex, and BMI, ADF remained independently associated with LS stage, indicating that a smaller ADF was associated with more severe LS. In contrast, HV and NH were not independently associated with LS after adjustment. Collectively, these findings suggest that, among the foot-related indices assessed, the mobility component—particularly ADF—may be more closely linked to LS severity.

A plausible mechanism underlying the association between limited ADF and LS is the restriction of the ankle rocker function during mobility and balance tasks. During the stance phase of gait and during sit-to-stand transitions, ankle dorsiflexion allows tibial progression, enabling forward movement of the center of mass within the base of support while maintaining stable support. When dorsiflexion is limited, tibial progression during stance is reduced, which may contribute to earlier heel rise, shortened step length, and decreased forward center-of-mass progression, thereby compromising gait efficiency and stability and necessitating increased foot stiffness [26]. Because passive ADF was measured with the knee fully extended to standardize gastrocnemius tension, the observed limitation likely reflects the extensibility of the gastrocnemius muscle–tendon unit. Clinically, tightness of this biarticular muscle can prematurely limit tibial advancement during mid-stance, thereby promoting early heel rise. In addition, across various gross motor tasks such as walking and rising from a chair, limited ADF may reduce the available “adjustment capacity” at the ankle, leading to greater trunk forward lean and increased reliance on hip and knee strategies (i.e., increased hip strategy), which may impair task stability [27,28,29]. Because LS encompasses declines in mobility-related functions including walking and standing up, constraints in movement strategies due to limited ADF may be reflected as greater LS severity.

The absence of independent associations for HV and NH should be interpreted in light of previous evidence and the interrelationships among foot indices. HV is not merely an isolated deformity but has been reported to relate to foot alignment, including reductions in the medial longitudinal arch, and co-occurrence of increased HV and lower arch height has been described [30]. Thus, when HV, NH, and ADF are entered simultaneously into the same model, shared information (i.e., common variance) across these indices may attenuate the independent contribution of HV and NH, while explanatory power may concentrate on an index that more directly constrains gross mobility (e.g., ADF). This overlap could make it more difficult to detect independent associations of HV or NH with LS.

Additionally, evidence regarding associations between foot indices (including HV) and mobility or gross motor performance has not been fully consistent across studies. While some studies report that HV is associated with impaired lower-limb function [31], others suggest no clear differences in dynamic balance indicators during walking among community-dwelling older adults [32]. The impact of HV on gait and balance may vary depending on the population, task conditions, and deformity severity. Although an HV angle of ≥20° is often classified as moderate [33], many participants in the present study had HV angles within the normal range, and even the LS3 group showed a median within the normal range. Therefore, relatively few participants may have had HV severe enough to meaningfully influence the functional measures, potentially limiting the ability to detect associations. Furthermore, the functional impact of HV may not be determined solely by angular deformity; it may emerge in combination with other factors such as toe function, sensory impairment, and pain [34]. Because the present study did not include pain, plantar sensation, or toe flexor strength as primary explanatory variables, the independent detectability of associations between HV or NH and LS may have been reduced. Similarly, NH is a static indicator of arch height and may not sufficiently represent functional properties such as load-bearing support, stiffness, or dynamic foot motion during gait [35,36]. Multicollinearity was not a concern in our models, suggesting that the non-significant independent associations for HV and NH were unlikely to be explained by collinearity.

We further explored whether physical function (gait speed, TUG, and SLS) could help explain the pathway linking foot indices to LS. When TUG or gait speed was added to the ordinal model, ADF remained inversely associated with LS severity, suggesting that the ADF–LS association is not fully explained by these physical function measures. SLS time was also significantly associated with LS stage. Although SLS time did not show a monotonic trend across LS stages in the unadjusted descriptive statistics (Table 1), the multivariable model indicated a significant inverse association between SLS time and LS stage. Importantly, in an additional model including ADF (minimum), SLS time, age, sex, and BMI, both smaller ADF and shorter SLS time were independently associated with a higher LS stage. Taken together, these findings indicate that limited ADF relates to LS severity beyond overall mobility (gait speed/TUG) and remains evident even after accounting for balance performance (SLS). This pattern suggests that balance-related constraints during single-limb support may be one pathway linking limited ADF to greater LS severity. One plausible explanation is that limited dorsiflexion constrains ankle strategy and lower-leg stabilization during single-limb support, potentially increasing instability during one-leg stance and thereby contributing to higher LS severity.

Consistent with this interpretation, partial correlation analyses adjusted for age, sex, and BMI demonstrated that ADF was associated with the two-step score, overall LS stage, and stand-up test performance level. Greater ADF was related to better performance on mobility and standing-up tasks and to less severe LS. These findings support the possibility that limited ADF contributes to LS severity through reduced physical performance, including mobility and standing-up capacity.

From a clinical perspective, the present results suggest that ADF may serve as a potential clinical marker associated with LS severity. Because LS is determined by multifactorial influences (e.g., strength, balance, pain, activity level, and comorbidities), poor LS stage or low performance on the two-step test, stand-up test, or GLFS-25 may arise from heterogeneous underlying mechanisms. Given that ADF was associated with two-step and stand-up performance in this study, when limited ADF is observed, clinicians may more readily formulate evaluation and intervention hypotheses that emphasize ankle mobility as a contributor to impaired mobility and rising performance. Although SLS is not a component of the LS test, it is widely used in clinical settings as a practical adjunct index of single-limb support ability; thus, assessing SLS alongside ADF may help contextualize the extent to which dorsiflexion limitation contributes to balance-related deficits.

Several limitations should be acknowledged. First, this cross-sectional design precludes causal inference; it remains unclear whether limited ADF increases LS severity or whether LS-related reductions in activity promote dorsiflexion limitation. Second, residual confounding is possible, including pain, medical history, physical activity, lower-limb muscle strength, and severity of joint disease. Third, HV and arch measures were based on surface-based assessments from photographs rather than radiographic or CT-based measurements; choices regarding representative values and measurement error may have influenced results. Because ADF was assessed passively in supine position, it may not fully reflect weight-bearing dorsiflexion during walking and balance tasks. In addition, because the knee was maintained in full extension during passive ADF measurement, the restriction may predominantly reflect gastrocnemius tightness rather than weight-bearing dorsiflexion capacity during functional tasks. Future studies should compare passive ADF with weight-bearing lunge measures and dynamic ankle kinematics. In addition, we did not evaluate intra- or inter-rater reliability for these photographic measures. Fourth, participants were recruited using convenience sampling of community volunteers, which may have introduced selection bias (e.g., individuals who are healthier or more health-conscious may have been more likely to participate) and may limit the generalizability of the findings to the broader older population. Therefore, the findings should be interpreted cautiously and require confirmation in larger, more representative cohorts. In addition, the distribution of LS stages was skewed, with relatively few participants in the higher LS stages, which may have limited statistical power for detecting small effects and for stage-specific comparisons. Future research should verify in longitudinal cohorts whether limited ADF predicts incident LS or the progression of LS severity. In addition, it will be important to statistically evaluate mediation pathways, such as whether limited ADF influences LS through balance impairment. Finally, intervention studies testing whether improving ADF can enhance LS-related outcomes and daily functioning would directly strengthen the clinical implications of these findings.

## 5. Conclusions

In community-dwelling older adults, ADF was independently associated with LS stage, such that smaller dorsiflexion was linked to more severe LS. Moreover, after adjustment for age, sex, and BMI, ADF was associated with performance on the two-step test and the stand-up test. These findings suggest that limited ankle dorsiflexion was associated with LS severity, potentially through reduced mobility and rising performance.

## Figures and Tables

**Table 1:** Participant characteristics, foot-related indices, and physical function measures by locomotive syndrome (LS) stage.

	Overall (n = 119)	LS Stage				
		LS 0 (n = 24)	LS 1 (n = 73)	LS 2 (n = 13)	LS 3 (n = 9)	*p* Value
Age (years)	76 [72–81]	75 [71–77]	76 [73–80]	77 [71–82]	82 [77–83]	0.051
Sex, n (%)	31 (26.1)	8 (33.3)	18 (24.7)	3 (23.1)	2 (22.2)	0.937
BMI (kg/m^2^)	22.8 [21.0–24.8]	22.1 [21.14–23.3]	22.7 [20.8–24.6]	24.5 [20.6–28.1]	24.3 [23.1–25.4]	0.159
HV angle (°)	16.0 [12.0–21.0]	15.5 [9.5–19.8]	16.0 [12.0–21.5]	16.0 [11.0–23.5]	18.0 [15.0–28.0]	0.484
NH (cm)	3.9 ± 0.6	3.8 ± 0.5	3.8 ± 0.6	4.1 ± 0.7	4.0 ± 0.6	0.614
ADF (°)	13.0 ± 7.2	15.4 ± 6.9 ^ab^	13.9 ± 7.1 ^cd^	8.0 ± 4.8 ^bd^	6.3 ± 6.0 ^ac^	<0.001 *
5 m gait speed (m/s)	1.3 [1.2–1.4]	1.4 [1.2–1.6] ^a^	1.3 [1.2–1.4] ^c^	1.1 [1.0–1.3]	1.0 [0.9–1.2] ^ac^	<0.001 *
TUG (s)	6.0 [5.4–7.0]	5.8 [5.3–6.2] ^ab^	5.9 [5.4–6.6] ^cd^	7.4 [6.5–8.5] ^bd^	8.0 [6.8–10.6] ^ac^	<0.001 *
SLS (s)	12.5 [4.6–30.8]	24.8 [6.4–60] ^ab^	13.0 [5.2–28.9]	24.6 [20.7–28.1] ^b^	24.3 [23.1–25.5] ^a^	<0.001 *

Values are presented as median [interquartile range] unless otherwise indicated. Navicular height and ankle dorsiflexion angle are presented as mean ± standard deviation. Sex is presented as males, n (%); females comprise the remainder. For bilateral measures, the maximum value was used for HV, and the minimum values were used for navicular height and ankle dorsiflexion. *p* values were calculated using one-way analysis of variance (ANOVA) for normally distributed continuous variables, the Kruskal–Wallis test for non-normally distributed continuous variables, and the chi-square test (or Fisher’s exact test when appropriate) for categorical variables. Post hoc pairwise comparisons were performed using Tukey’s test (after ANOVA) or Dunn’s test (after Kruskal–Wallis), with multiplicity adjusted as appropriate (e.g., Holm correction); superscripts denote significant pairwise differences (adjusted *p* < 0.05) as follows: ^a^, LS 0 vs. LS 3; ^b^, LS 0 vs. LS 2; ^c^, LS 1 vs. LS 3; ^d^, LS 1 vs. LS 2; letters are shown for both groups involved in each significant comparison. Abbreviations: LS, locomotive syndrome; BMI, body mass index; HV, hallux valgus; NH, navicular height; ADF, ankle dorsiflexion angle; TUG, Timed Up and Go; SLS, single-leg stance. * *p* < 0.001.

**Table 2:** Ordinal logistic regression for locomotive syndrome stage (0–3).

Predictor	OR (per 1 Unit)	95% CI	*p* Value
Age (years)	1.08	0.99–1.17	0.082
Sex (male vs. female) ^†^	0.42	0.15–1.16	0.092
BMI (kg/m^2^)	1.15	1.01–1.30	0.038 *
HV (°)	1.03	0.97–1.09	0.28
NH (cm)	1.26	0.57–2.77	0.573
ADF (°)	0.91	0.85–0.98	0.008 **

Model fit: LR χ^2^(6) = 20.53, *p* = 0.002; residual deviance = 186.72; AIC = 204.72. Proportional-odds ordinal logistic regression was used with locomotive syndrome stage (0–3) as the dependent variable. Odds ratios (ORs) represent the change in the odds of being classified into a higher (more severe) LS stage per 1-unit increase in each predictor. Sex was coded as 0 = female and 1 = male; thus, the OR for sex indicates male (vs female). For bilateral measures, the maximum value was used for hallux valgus angle (HV), whereas the minimum values were used for navicular height (NH) and ankle dorsiflexion (ADF) as representative values. *p* values were obtained from Type II likelihood-ratio tests. Abbreviations: ADF, ankle dorsiflexion; BMI, body mass index; CI, confidence interval; HV, hallux valgus; NH, navicular height. ^†^ Reference category: female. Statistical significance is indicated as * *p* < 0.05, ** *p* < 0.01.

## Data Availability

The datasets generated and/or analyzed during the current study are available from the corresponding author on reasonable request.

## Supplementary Materials

The following supporting information can be downloaded at: https://www.mdpi.com/article/10.3390/healthcare14060742/s1, Supplementary Table S1. Extended ordinal logistic regression models for LS stage (0–3). Supplementary Table S2. Binary logistic regression for Locomotive Syndrome (LS) stage ≥ 2 (vs. <2). Supplementary Table S3. Partial Spearman correlations (adjusted for age, sex, and BMI).
