# Supplementary material for: Passive Ankle Dorsiflexion and Single-Leg Balance Are Independently Associated with Locomotive Syndrome Severity in Community-Dwelling Older Adults: A Cross-Sectional Study
Source: Healthcare (Basel). 2026 Mar 14;14(6):742. doi: 10.3390/healthcare14060742 (PMC13027063; doi:10.3390/healthcare14060742)
Supplement: Supplementary file 1 [file healthcare-14-00742-s001.zip › Supplementary Table S1.pdf]

Supplementary Table S1. Extended ordinal logistic regression models for LS stage (0–3).

Predictor	Primary model (Foot indices + age, sex, BMI)	Extended model + TUG	Extended model + gait speed	Extended model + SLS
BMI (per 1 kg/m²)	1.15 (1.01–1.30), 0.038	1.02 (0.89–1.18), 0.748	1.12 (0.98–1.27), 0.092	1.12 (0.98–1.27), 0.101
HV angle (per 1°)	1.03 (0.97–1.09), 0.280	1.02 (0.96–1.08), 0.522	1.03 (0.97–1.09), 0.391	1.05 (0.99–1.11), 0.128
ADF (per 1°)	0.91 (0.85–0.98), 0.008	0.92 (0.86–0.99), 0.015	0.93 (0.87–1.00), 0.047	0.93 (0.86–0.99), 0.025
NH (per 1 mm)	1.26 (0.57–2.77), 0.573	1.51 (0.67–3.40), 0.324	1.50 (0.66–3.40), 0.327	1.59 (0.70–3.63), 0.266
Sex (male vs female)	0.42 (0.15–1.16), 0.092	0.27 (0.09–0.80), 0.016	0.32 (0.11–0.93), 0.034	0.34 (0.12–0.99), 0.045
Age (per 1 year)	1.08 (0.99–1.17), 0.082	1.03 (0.95–1.12), 0.492	1.06 (0.97–1.15), 0.205	1.06 (0.97–1.15), 0.184
TUG (per 1 s)	—	2.34 (1.48–3.70), <0.001	—	—
Gait speed (per 0.1 m/s)	—	—	0.75 (0.59–0.97), 0.023	—
SLS (per 1 s)	—	—	—	0.97 (0.95–0.99), 0.009
Model fit vs intercept-only (LR chi-square, df, p)	LR $\chi^2(6)=20.53$; $p=0.002$	LR $\chi^2(7)=34.97$; $p<0.001$	LR $\chi^2(7)=25.73$; $p<0.001$	LR $\chi^2(7)=27.36$; $p<0.001$
AIC	204.72	192.27	201.52	199.89
Residual deviance	186.72	172.27	181.52	179.89
Proportional odds assumption (LRT vs multinomial, p)	0.675	0.119	0.454	0.651

Values are odds ratios (ORs) with 95% confidence intervals (CIs) from proportional-odds ordinal logistic regression for LS stage (0–3), interpreted as the odds of being in a higher LS stage. Foot indices were derived from bilateral measures (HV angle, NH, ADF). Sex was coded 0=female and 1=male. Extended models added one physical function measure (TUG, gait speed, or SLS) to the primary model. Gait speed is presented per 0.1 m/s; other continuous predictors are per 1 unit (BMI per 1 kg/m²; angles per 1°; navicular height per 1 mm; age per 1 year; times per 1 s). Model fit is shown by LR tests vs intercept-only, AIC, and residual deviance; the proportional odds assumption was assessed by LR testing against multinomial models. Two-sided p values are shown; complete-case analyses.
