# Supplementary material for: Passive Ankle Dorsiflexion and Single-Leg Balance Are Independently Associated with Locomotive Syndrome Severity in Community-Dwelling Older Adults: A Cross-Sectional Study
Source: Healthcare (Basel). 2026 Mar 14;14(6):742. doi: 10.3390/healthcare14060742 (PMC13027063; doi:10.3390/healthcare14060742)
Supplement: Supplementary file 1 [file healthcare-14-00742-s001.zip › Supplementary Table S2.pdf]

Supplementary Table S2. Binary logistic regression for Locomotive Syndrome (LS) stage ≥2 (vs <2).

Predictor	β	SE	OR	95% CI (lower)	95% CI (upper)	p value
HV (per 1°)	-0.006	0.033	0.994	0.929	1.061	0.854
ADF (per 1°)	-0.163	0.053	0.849	0.757	0.935	0.002
NH (per 1 mm)	0.383	0.519	1.466	0.529	4.142	0.461
BMI (per 1 kg/m²)	0.146	0.082	1.157	0.983	1.368	0.076
Sex (1=male)	-1.275	0.752	0.28	0.057	1.121	0.09
Age (per 1 year)	0.046	0.052	1.047	0.948	1.165	0.376

The outcome was dichotomized as Locomotive Syndrome (LS) stage ≥2 (coded as 1) versus LS stage <2 (coded as 0). Predictors were prespecified "worst-side" representative values (HV, NH and ADF). Sex was coded as 0=female and 1=male. Analyses were conducted using complete cases (n=117). Model fit: likelihood-ratio test versus the intercept-only model, $\chi^2(6)=24.87$, $p<0.001$; Hosmer–Lemeshow goodness-of-fit test, $\chi^2(8)=2.19$, $p=0.975$. Variance inflation factors ranged from 1.065 to 1.431.
