# Supplementary material for: Passive Ankle Dorsiflexion and Single-Leg Balance Are Independently Associated with Locomotive Syndrome Severity in Community-Dwelling Older Adults: A Cross-Sectional Study
Source: Healthcare (Basel). 2026 Mar 14;14(6):742. doi: 10.3390/healthcare14060742 (PMC13027063; doi:10.3390/healthcare14060742)
Supplement: Supplementary file 1 [file healthcare-14-00742-s001.zip › Supplementary Table S3.pdf]

Supplementary Table S3. Partial Spearman correlations (adjusted for age, sex, and BMI)

Variable pair	ρ	p value	df
DF vs 2-step test score	0.246	0.008*	112
DF vs GLFS-25 score	-0.312	0.001**	112
DF vs Stand-up test stage	0.334	0.001**	112

Function Scale; df, degrees of freedom; BMI, body mass index.

Note: Partial correlations are reported as partial Spearman's rank correlation coefficients (ρ) controlling for age, sex, and BMI.

(*): $p < 0.01$, (**): $p < 0.005$

Abbreviations: DF, ankle dorsiflexion angle (°); GLFS-25, 25-question Geriatric Locomotive
